# Malaria in East African highlands during the past 30 years: impact of environmental changes

**DOI:** 10.3389/fphys.2012.00315

**Published:** 2012-08-02

**Authors:** Yousif E. Himeidan, Eliningaya J. Kweka

**Affiliations:** ^1^Entomology Unit, Faculty of Agriculture and Natural Resources, University of KassalaNew Halfa, Sudan; ^2^Mosquito Section, Division of Livestock and Human Health Vector Control, Tropical Pesticides Research InstituteArusha, Tanzania

**Keywords:** malaria, East African highlands, environmental changes

## Abstract

East African highlands are one of the most populated regions in Africa. The population densities in the highlands ranged between 158 persons/km^2^ in Ethiopia and 410 persons/km^2^ in Rwanda. According to the United Nations Population Fund, the region has the world's highest population growth rate. These factors are likely behind the high rates of poverty among the populations. As there were no employment opportunities other than agricultural, this demographic pressure of poor populations have included in an extensive unprecedented land use and land cover changes such as modification of bushland, woodland, and grassland on hillsides to farmland and transformation of papyrus swamps in valley bottoms to dairy pastures and cropland and changing of fallows on hillsides from short or seasonal to longer or perennial. Areas harvested for food crops were therefore increased by more than 100% in most of the highlands. The lost of forest areas, mainly due to subsistence agriculture, between 1990 and 2010 ranged between 8000 ha in Rwanda and 2,838,000 ha in Ethiopia. These unmitigated environmental changes in the highlands led to rise temperature and optimizing the spread and survival of malaria vectors and development of malaria parasites. Malaria in highlands was initially governed by low ambient temperature, trend of malaria transmission was therefore increased and several epidemics were observed in late 1980s and early 2000s. Although, malaria is decreasing through intensified interventions since mid 2000s onwards, these environmental changes might expose population in the highlands of east Africa to an increase risk of malaria and its epidemic particularly if the current interventions are not sustained.

## Geography and climate of east african highlands

**Figure 1:**
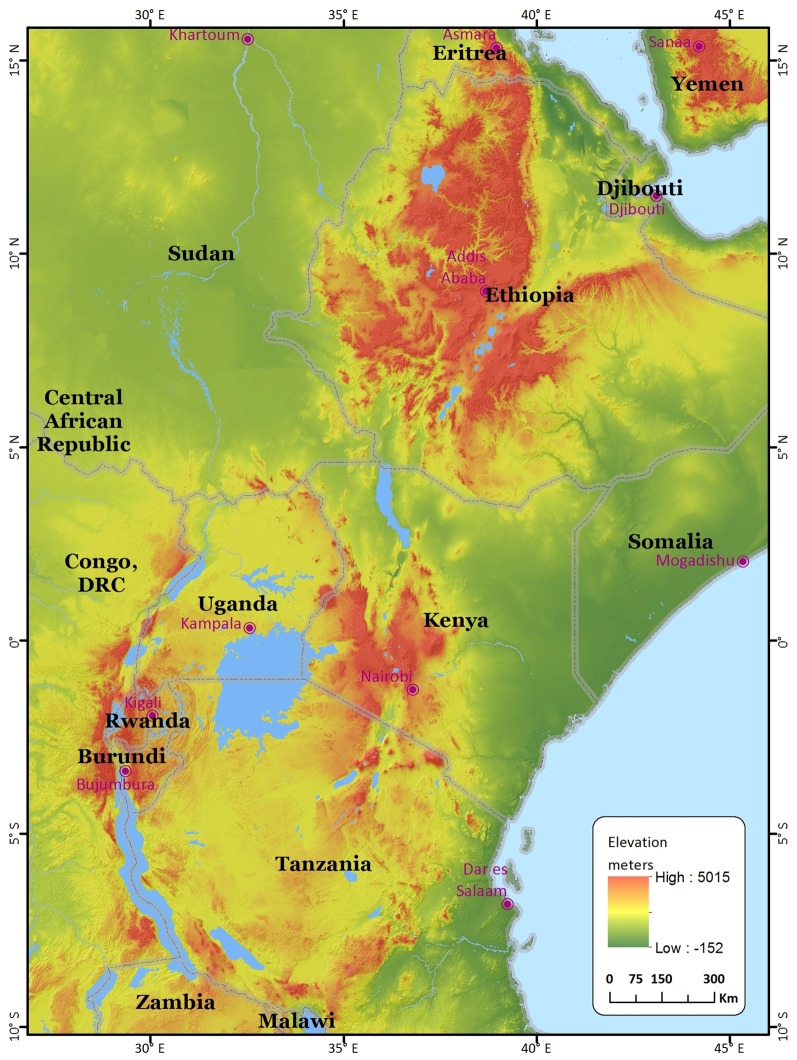
**Highlands areas in East African countries**.

In Africa, the highlands are defined to be altitude higher than 1500 m elevation above sea level or with daily mean temperatures of below 20°C. This area covers about one million km^2^ equivalent to 4% of the total land area of sub-Saharan region. Ethiopia, Kenya, Tanzania, Rwanda, Burundi, Uganda, and Madagascar constitute about 82.4% of all African highlands. Ethiopian highlands make up 60% of all highlands in East Africa, while other countries constitutes of variable proportions of the remainder (Figure 1) (Hurni, 1990). The highlands of East Africa have been endowed with a combination of moderate temperatures, adequate rainfall (falling in two distinct seasons for much of the highlands), and productive soils that make the region one of the best suitable regions for agricultural development in Africa.

## Populations of east african highlands

Although the highland zone is a small fraction of the total land area of sub-Saharan Africa, it accounts for large populations of human and livestock due to its high agricultural productivity potential (Getahun, 1978). In general, East African highlands are considered as the most populated parts of the region (Bloom and Sachs, 1998). Despite the fact that the highland constitutes 23% of the total landmass in the region, their human dwellings represent more than 64% of its population with a population densities ranged between 158 persons/km^2^ in Ethiopia and 410 persons/km^2^ in Rwanda (Table 1). Densities above 500 persons/km^2^ is common in some parts of the highlands i.e., the population densities in Kigezi District, the southwestern highlands of Uganda, is 518 persons/km^2^ (Bolwing, 2006) and it is exceed to 1200 persons/km^2^ in Vihiga district, western highlands of Kenya (Delve and Ramisch, 2006). The United Nations Population Fund (Formerly- United Nations Fund for Population Activities or UNFPA) data showed that Eastern Africa region already in 2010 having advanced into the most populated region in Africa and still has the world's highest population growth rate (UNFPA, 2010).

**Table 1:** **Population density in East African highlands**.

**Country**	**Population in 1985**	**Population in 2005**	**Population in 2010**	**Population growth 2005–2010**	**Population density**	**Total area in highlands (km^2^)**	**Population in highlands (%)**	**Population density in highlands**
Burundi	4922	8162	9863	3.90	298	23,182	82.1	349
Ethiopia	42,227	74,980	88,013	2.51	72	489,500	88.0	158
Kenya	19,761	34,912	40,047	2.65	69	128,300	82.5	257
Madagascar	10,029	18,312	21,282	2.66	28	16,825	26.2	331
Rwanda	5987	9611	11,056	2.76	380	25,918	96.2	410
Tanzania	21,618	37,771	41,893	2.47	46	119,600	61.4	215
Uganda	14,392	28,199	33,399	3.24	136	20,000	16.0	268

Source: The population and population growth rate is based on the estimates taken from the 2006 edition of the United Nations World Population Prospects report. Population density figures are sourced from year 2005 data in United Nations World Population Prospects (2004 revision).

Agriculture is the main livelihood for the populations of East African countries i.e., more than 85, 80, and 75% of the total labor force is engaged in agriculture in the highlands of Ethiopia, Uganda, and Kenya, respectively. Although the highlands include the most favorable agricultural production areas, the populations are characterized by disappointingly high rates of poverty and one of the reasons behind this is the extreme population density (Place et al., 2006c). In Kenya, for example, about 53–56% of the population lives below the Kenyan poverty line of $0.55/day in central and western Kenya highlands (Pell et al., 2004). Unfortunately, non-agricultural employment opportunities are not growing rapidly enough to provide the engine for a viable poverty reduction strategy for the short to medium term (Place et al., 2006b).

## History and current situation of malaria in east african highlands

**Figure 2:**
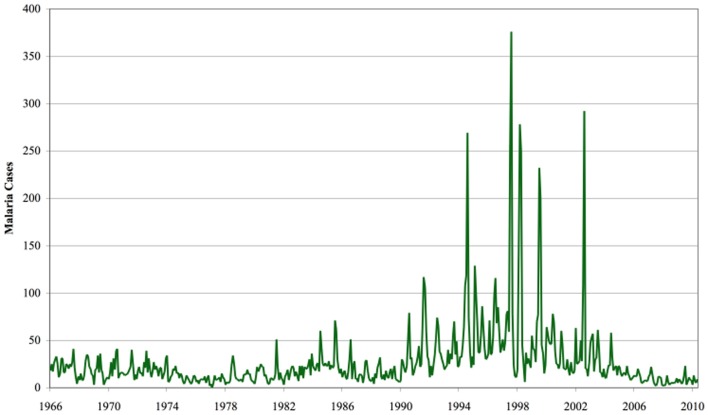
**Monthly malaria cases at Kericho Unilever Tea Kenya Ltd. Hospital**. Source: Stern et al. (2011), with permission from Dr. David I. Stern, Crawford School of Economics and Government, Australian National University.

During nineteenth century, highland areas have been considered to be free or negligible malaria incidence (Matson, 1957; Lindsay and Martens, 1998). Movement of people to lowlands associated with the opening of civil and military posts, increased agricultural activities probably introduced malaria into the highlands (Malakooti et al., 1998; Shanks et al., 2005). The first malaria epidemic was documented following the influenza pandemic during troop demobilization and resettlement after World War I in 1918 and 1919 in western Kenya highlands (Matson, 1957). Between the 1920s and the 1950s infrequent malaria epidemics were reported in eastern Africa highlands (Fontaine et al., 1961). Malaria epidemics were not reported between the 1960s and the early 1980s after a malaria eradication campaign (Roberts, 1964a,b,c). Records from tea estates in the Kericho district of western Kenya highlands showed that malaria re-emerged in the 1980s (Shanks et al., 2005) (Figure 2). Thereafter, several malaria epidemics were reported during the decades of 1980s and 1990s in most countries of eastern Africa highlands. These include Uganda (Mouchet, 1998), Kenya (Some, 1994; Malakooti et al., 1998; Lindblade et al., 2000a), Ethiopia, Tanzania (Matola et al., 1987; Fowler et al., 1993; Mboera and Kitua, 2001), Rwanda (Loevinsohn, 1994), and Madagascar (Lepers et al., 1988; Mouchet, 1998). In western Kenya highlands alone between late 1980s and early 2000s, malaria outbreaks were reported at more than 20 highland sites causing serious mortality and morbidity (Lepers et al., 1988; Loevinsohn, 1994; Some, 1994; Lindsay and Martens, 1998; Mouchet, 1998; Shanks et al., 2000; Hay et al., 2002a,b).

The patterns of malaria epidemic during the 1990s were characterized by expanded geographic areas, increased frequencies and case-fatality rates (Githeko and Ndegwa, 2001; Zhou et al., 2004; Shanks et al., 2005; Pascual et al., 2008). Both the frequency of malaria epidemics and the number of malaria outpatients have dramatically increased compared to those in the 1980s such as in Kericho (Figure 2) (Snow et al., 1999; Shanks et al., 2005). Compared to the 1960s, case fatality rate increased from 1.3% to 6% in the 1990s (Shanks et al., 2000). A significant human mortality in both children and adults was reported during the last malaria epidemics in 2002 and 2003 in Kericho and Nandi districts of western Kenya highlands (Hay et al., 2002b,^2003^; John et al., 2004; Shanks et al., 2005). The estimates of WHO in 2003 showed that malaria epidemics kill 110,000 people each year and 110 million people were considered at risk in the highlands (WHO, 2003).

A complex set of environmental, biological, and socioeconomic factors including climate change (Loevinsohn, 1994; Lindsay and Martens, 1998; Afrane et al., 2006), land use changes (Matola et al., 1987; Lindsay and Martens, 1998; Malakooti et al., 1998; Mouchet, 1998), drug resistance (Malakooti et al., 1998), cessation of malaria control activities (Mouchet, 1998), and demographic changes (Lindsay and Martens, 1998; Mouchet, 1998) were hypothesized to influence malaria in the highlands. Apparently, a significant environmental change occurred. This was attributed mainly to dramatic increases in the human population i.e., populations of each of the East African countries increased by more than 100% since 1985 (Table 1), and this included an extensive unprecedented land use and land cover changes (Whitmore, 1997).

## Land cover change and its impact on malaria transmission

There is no doubt that population pressure in eastern African highlands profoundly changed land cover over the course of the last one or two centuries, and even earlier than that (Bolwing, 2006). Data presented in Table 2 shows that lost of forest areas during 1990–2010 in the countries mentioned of east Africa due to human activities was ranged between 8000 ha in Rwanda and 2,838,000 ha in Ethiopia. Recent study from Madagascar indicated that about 88.91 km^2^ was shrunk only between 2003 and 2004 of forest near Ranomafana National Park (Brooks et al., 2009). This occurred mainly due to increased human demand for forest products and land for agricultural purposes. In Rwanda, deforestation of the entire country is almost completely due to overpopulation, with a mere 230 square miles (600 km^2^) remaining and an ongoing loss of about 9% per annum (McMichael, 2003). In highlands of Kenya, reduction in tree cover was found in areas with more densely populated (Pell et al., 2004). The rapid loss of primary forest due to subsistence agriculture (Myers, 1979, 1984; Brooks et al., 2009) represents one of the greatest environmental changes that create disequilibrium to local natural balance and global biodiversity. It was concluded that unmitigated deforestation under increasing demographic pressure make the East African highlands one of the most fragile ecologies in the world (McMichael, 2003).

**Table 2:** **Trends in natural forest cover (1000 ha) (Deforestation) in East African, 1990–2010**.

**Country**	**Natural forest cover (1000 ha)**				**Loss (1000 ha)**	**Coverage %**	**Annual deforestation rate (%)**		
	**1990**	**2000**	**2005**	**2010**	**1990–2010**	**2000**	**1990–2000**	**2000–2005**	**2005–2010**
Burundi	289	112	103	103	186	3.7	−6.1	−6.12	−0.8
Ethiopia	14,623	13,214	12,509	11,785	2838	4.2	−1.0	−0.96	−1.08
Kenya	3470	3370	3320	3270	200	30.0	−0.3	−0.29	−0.30
Madagascar	13,461	12,850	12,548	12,138	1323	20.2	−0.5	−0.45	−0.55
Rwanda	70	62	62	62	8	n.a	−1.1	−1.14	0
Tanzania	41,345	37,262	35,215	33,188	8157	43.9	−1.0	−0.99	−1.09
Uganda	4717	3837	3398	2937	1780	21.0	−1.9	−1.87	−2.35

Data source: Rhett A. Butler/mongabay.com. San Francisco, USA. Available at: http://rainforests.mongabay.com/deforestation/ Retrieved on January 20, 2012

The implication of this change in land cover on malaria transmission is that deforestation can lead to changes in microclimate of both adult and larval habitats, hence increase larvae survival, population density, and gametocytes development in adult mosquitoes (Afrane et al., 2006; Munga et al., 2006, 2007; Kweka et al., 2011). For example, the mean indoor temperatures of houses located in the deforested area of western Kenya highland were found to range between 0.7°C and 1.2°C higher than in houses located in the forested area, which resulted in a significant increase in net reproductive rate and intrinsic growth rate for adult mosquitoes (Afrane et al., 2006). This enhanced mosquito reproductive fitness and mosquito population growth, shortened the duration of the gonotrophic cycles by 1.4–1.5 days. Deforestation could augment the vectorial capacity of *Anopheles gambiae* with 29–106% increase compared with forestation areas (Afrane et al., 2005, 2006). Deforestation was found to increase water temperature of larval habitat, hence increase immature stages survivorship of malaria vectors by shortening larval development period and reducing the chance of larvae encountering predators (Tuno et al., 2005; Munga et al., 2007). Not only, changing microclimate of both larval and adult stages of malaria vectors, and development of malaria parasite, but also deforestation was shown to create more suitable breeding sites (Minakawa et al., 2005; Munga et al., 2005; Mushinzimana et al., 2006). Forest reclamation for agriculture, was found to change water chemistry which is suitable for larval development and survival (Munga et al., 2005; Tuno et al., 2005). Therefore, the overall consequences of deforestation, particularly due to expansion of agricultural activities can increase malaria cases and transmission e.g., in western Usambara forest (Bodker et al., 2003, 2006).

## Land use change and its impact on malaria transmission

In more humid highlands area having bimodal rainfall patterns and sufficiently good soils, production of perennial cash crops such as coffee is common e.g., highlands of central Kenya (Place et al., 2006c), Eastern highlands of Uganda (Pender et al., 2006), and much of southwestern Ethiopia. Perennial food crops are also common in such areas, but annual food crops (especially maize) are become more important in many areas, particularly in western Kenya highlands, where maize–livestock production is the dominant farming system (Place et al., 2006b).

There is no doubt that population pressure in the region led to expanded and intensified agricultural activities and pastoral land use systems as a response to increasing population densities and market opportunities (Bolwing, 2006). For example, the increase in area harvested for only one single crop of maize ranged between 4.7% in Burundi and 225.7% in Rwanda (Table 3). In the southwestern “Kigezi” highlands of Uganda, for example, remotely sensed land cover data from the early 1990s show that small scale farmland covers 57% and 68% of the land area in Kabale and Kisoro districts respectively, while natural forests cover only 2.0% of Kabale and 16.3% of Kisoro (Bolwing, 2006). During the last four decades, in the Bale Mountains in southeast Ethiopia the area changed from a quite natural to a more cultural landscape. Within a representative subset of the study area (7957.5 km^2^), agricultural fields increased from 1.71% to 9.34% of the total study area since 1973 (Kidane et al., 2012). Cultivation of valley papyrus swamps around Lake Bunyonyi produced major changes in land uses (Lindblade et al., 2000b). This activities of claiming natural swamps for cultivation started more than 70 years ago when the British administration encouraged the drainage of swamps containing *Cyperus papyrus* (papyrus) and other swamp grasses to increase the land available for crop cultivation (Carswell, 1996). Of an estimated 69.7 km^2^ of papyrus swamp surveyed in 1954–1955 in Kabale District, south-western highlands of Uganda, only about 15% remains today in its natural state (Lindblade et al., 2000b). Bolwing (2006) summarized the changes in land use between the 1950s and 1990s in the highlands of East Africa where; bushland, woodland, and grassland on hillsides was changed to farmland; papyrus swamps in valley bottoms was changed to dairy pastures and cropland; grazing land on steep slopes have been changed to fallow areas and short (seasonal) fallows on hillsides have been changed to longer fallows on hillsides.

**Table 3:** **Increase in harvested area of maize in East Africa**.

**Country**	**Total area in highlands (km^2^)**	**% of African highlands^a^**	**Area harvested (ha) 1980**	**Area harvested (ha) 1995**	**Area harvested (ha) 2010**	**% increase in area harvested**
Burundi	23,182	2.6	1,300,00	1,200,000	1,256,000	4.7
Ethiopia	489,500	49.2	n.a.	1,464,080	1,772,250	21.0
Kenya	128,300	12.9	1,350,000	1,438,740	2,008,350	45.8
Madagascar	16,825	1.2	1,278,900	1,838,400	3,712,000	132.3
Rwanda	25,000	2.5	718,000	500,000	1,846,580	225.7
Tanzania	119,600	12.0	1,400,000	1,368,000	3,100,000	124.3
Uganda	20,000	2.0	258,000	571,000	890,000	110.7

a Other non eastern African highlands represent 17.6%.

Source: Food and Agriculture Organization (FAO), FAOSTAT, Available at: http://faostat.fao.org/DesktopDefault.aspx?PageID=567#ancor. Retrieved on 23 January 2012.

Data for total area in highlands (km^2^) were obtained from Jahnke, H.: “Dairy development in the highlands of tropical Africa: an overview of planning considerations”; paper presented at the International Livestock Centre for Africa, Workshop on Smallholder Dairy Development in the East African Highlands, August 1980. Report is available at: http://www.ilri.org/InfoServ/Webpub/fulldocs/Bulletin11/Smallholder.htm#TopOfPage Retrieved on 20 January 2012.

**Figure 3:**
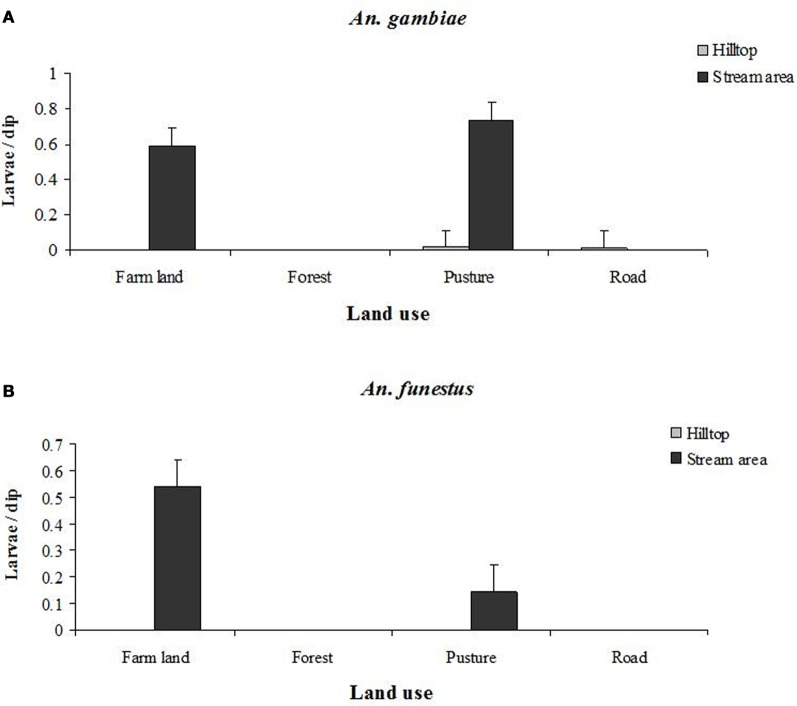
**Larvae density (larvae/dip) of *A. gambiae* (A) and *A. funestus* (B) among the different land use types found in Iguhu area in western Kenya highlands**. Error bars show standard errors. Source: Himeidan (2009).

These changes in land use were reported to create and spread habitats for malaria vectors breeding as well as changing microclimate by altering temperature to that more suitable for larval development and adult survival (Lindblade et al., 2000b). Some earlier reports noted that anophelines would not breed in papyrus swamps but could be found in the ditches formed during cultivation (Goma, 1958). Fifty years later, it was confirmed that *A. gambiae* and *Anopheles funestus* were the dominated farmland areas of western Kenya highlands (Figure 3) (Minakawa et al., 2005; Munga et al., 2006; Himeidan, 2009). Entomological surveys revealed that at least more than third of malaria vectors larval habitats observed in farmlands. These habitats constituted 40% of positive anopheline larval habitats (Minakawa et al., 2005), but almost 100% of habitat produce the vectors with rate of 1.82 *A. gambiae* s.l. emerged/m^2^/week compared to zeros in forests and swamps of western Kenya highlands (Munga et al., 2006). The successful of these habitats for producing the vectors was associated with the increase in maximum temperature of water of the breeding sites which was 6.6°C and 2.4°C higher in farmlands compared to forest and natural swamps, respectively (Munga et al., 2006). Lindblade and others (Lindblade et al., 2000b) compared mosquito density, biting, sporozoite, and entomological inoculation rates between eight villages located along natural papyrus swamps and eight villages located along swamps that drained and cultivated in Kabale District, south-western highlands of Uganda. They observed that both mean maximum and minimum temperatures were approximately 0.9°C higher in villages located along cultivated swamps than in villages located along natural swamps and that all malaria indices investigated were higher near cultivated swamps. The authors concluded that replacement of natural swamp vegetation with agricultural crops led to increase temperatures, which might be responsible for elevated malaria transmission risk in cultivated areas. This supported by the fact that several malaria outbreaks occurred near cultivated swamps and that was attributed to availability of potential breeding habitats for *Anopheles* vectors in highlands (Steyn, 1946; Goma, 1958). Overall, external environmental influences of household associated by land use changes such as living on flat land, in close proximity to maize fields, and on land lacking nearby trees were shown to increase malaria risk in East African highlands (Ernst et al., 2009).

## Impact of land use and land cover changes on malaria vectors distribution

Malaria vectors for many years were found in lowlands and restricted to highlands because of climatic conditions with low temperatures and high forest canopy (Githeko et al., 2000). Deforestation and land use changes created both temporal and permanent potential breeding habitats exposed to sunlit hence increase larvae survivorship and growth rate (Minakawa et al., 2004, 2005; Munga et al., 2005, 2006). The high rate of deforestation leads to rise of temperatures in highlands area (Githeko and Ndegwa, 2001; Afrane et al., 2005). Therefore, due to these changes, malaria vector mosquitoes (*A. gambiae* s.s and *A. funestus*) invaded the highland areas in western Kenya (Zhou et al., 2004; Ndenga et al., 2006). *Anopheles arabiensis* is highest vector ever reported and continue to re-colonize new areas in highlands of Kenya (Chen et al., 2006; Imbahale et al., 2011; Kweka et al., 2011). The high proportions of new species in East African highlands might increase the risk in malaria foci expansion and subsequently epidemics due to low immunity against malaria parasites among highland populations (Lindsay and Martens, 1998).

## Regional climate change and malaria epidemic

**Figure 4:**
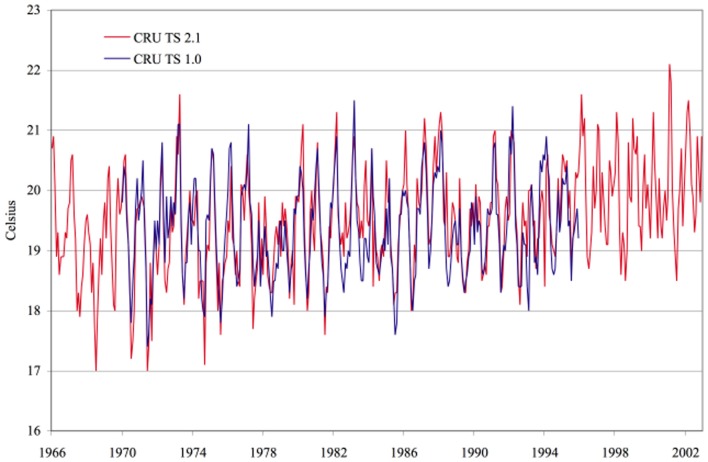
**Temperature series for Kericho: CRU TS 1.0 vs. CRU TS 2.1**. Source: Stern et al. (2011), with permission from Dr. David I. Stern, Crawford School of Economics and Government, Australian National University.

**Figure 5:**
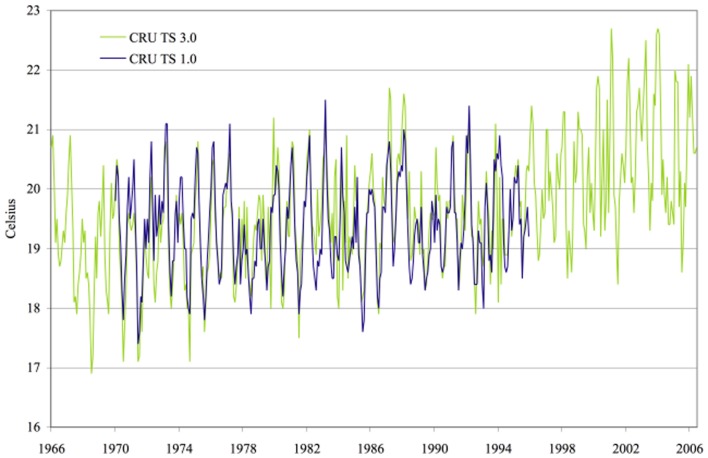
**Temperature series for Kericho: CRU TS 1.0 vs. CRU TS 3.0**. Source: Stern et al. (2011), with permission from Dr. David I. Stern, Crawford School of Economics and Government, Australian National University.

Both land use/land cover changes and global warming may contribute to regional change in climate of East African highlands. The presence of greenhouse gases in the atmosphere is the best known impact of human activity on climate change, variations in land use/land cover, and surface cover may be of equal importance (Pielke, 2005). In a transient climate simulation, agricultural expansion results in significant additional warming over the Amazon (Feddema et al., 2005). Though, as malaria in highlands is limited by low temperature (Hay et al., 2002b; Balls et al., 2004), regional climate changes have been proposed as a major factor accountable for the recent epidemics in African highlands (Martens, 1995; Lindblade et al., 2000b). However, assessing the impact of climate in malaria resurgence is difficult due to high spatial and temporal climate variability and the lack of long-term data series on malaria cases. Therefore, association between climate change and the re-emergence of malaria epidemic in the East African highlands is subject of debates during the last two decades. However, different observations were reported on climate change and their association with malaria incidence in African highlands. Despite an increase of 0.74°C, in the global average temperature between the years 1906 and 2005 (IPCC, 2007), Hay et al. (2002a) claimed that mean temperature and rainfall did not change significantly in the past century at four locations in the East African highlands, where malaria incidence increased. Patz et al. (2002) argued this conclusion to the use of spatially interpolated climate data that criticized for its inappropriateness for trend analysis in areas known to have a high spatial heterogeneity in temperature. The primary argument here was that climate was the main driver behind higher malaria incidence, but that its role could not be ruled out on the basis of lack of evidence for temperature warming in the region (Pascual et al., 2006). Based on this assumption, Pascual et al. (2006) revisited result of Hay et al. (2002b) using the same temperature data, with updated from 1950 to 2002 and found evidence for a significant warming trend at all four studied sites. Chaves and Koenraadt's (2010) assessed the conclusions from both sides of the argument that supporting and rebutting the role of climatic change as a driving force for highland invasion by malaria and concluded that evidence for the role of climate in these dynamics was robust. This conclusion was supported by Omumbo et al. (Myers, 1979) who analyzed quality controlled daily observations (>97% complete) of maximum, minimum, and mean temperature at Kericho meteorological station, sited in a tea growing area of Kenya's western highlands for 30 years (1 January 1979–31 December 2009). Evidence of a warming trend was also confirmed in this analysis and an upward trend of ≈0.2°C/decade was observed in maximum, minimum, and mean temperatures at Kericho in western Kenya highland (Chaves and Koenraadt, 2010; Omumbo et al., 2011; Chaves et al., 2012). Stern et al. (2011) compared a new robust trend test to the original monthly time series from the Climate Research Unit Time Series (CRU TS) 1.0 data set used by Hay et al. (Fowler et al., 1993) for the four locations in highland East Africa to the more recently published CRU TS 2.1 data used by Chaves and Koenraadt (2010) and Pascual et al. (2008) and to a newest data-set, CRU TS 3.0 as well as the data from the Kericho meteorological station used by Omumbo et al. (Myers, 1979). The authors confirmed the significant trends observed in the data extracted from newer editions of the database used by Chaves and Koenraadt (2010) and Pascual et al. (2008). The trend was also significant in the newest data-set, CRU TS 3.0 and in the data of the Kericho meteorological station but not in the older version (CRU TS) 1.0 for periods ending in 1996 used by Hay et al. (2002a) (Figures 4, 5). The role of this unambiguous warming trend observed on malaria transmission may need to be assessed again but indications provided in this review suggest that in this region, change of 0.7–1.2°C in temperature can have a significant effect on transmission (Afrane et al., 2005, 2006). Overall, it has been projected that climate changes will significantly affect the spread of malaria in tropical Africa well before 2050 and that the geographic distribution of areas where malaria is epidemic e.g., highlands might have to be significantly altered in the coming decades (Ermert et al., 2012).

## Complexity of highlands malaria transmission

The fact is that the occurrence of increased malaria transmission trends observed during 1980s and 1990s was not homogeneous across the East African highlands (Chaves et al., 2012). Topography synergize with increase rainfall associated with El Niño (Myers, 1984; Mboera and Kitua, 2001; Bodker et al., 2003; Kweka et al., 2011; Chaves et al., 2012), interaction between rainfall and temperature (Zhou et al., 2005), were major drivers of epidemics confirmed above 1600 m (Chaves et al., 2012). Although, shared biophysical environments can produce clusters of higher transmission, other factors at the individual and household levels can mediate this risk (Ernst et al., 2009). In this context, migration of human derived earlier by colonies administration; for settlement the highlands that favored by rich soil, maximum water resources and lower incidence of human diseases, or occurring later to the region associated with rapid population growth rate is probably playing role during both earlier emergence and the resurgence of malaria epidemics in the East African highlands (McCallum et al., 2001). This is probably indirect role occurred through a large proportion of land use changes that have been observed on a nearly continuous basis for many decades if not centuries (Place et al., 2006a). Forests and natural swamps of the highlands have mostly disappeared, and locals are still clearing the last patches of forest and drained the remaining natural swamps (Lindblade et al., 2000b; Verschuren et al., 2002), just for their self interest of increasing land available for agriculture (Ernst et al., 2009). It is believed that even there are no additional productive lands to be brought under cultivation and therefore, to reduce the poverty, agricultural growth must be through intensification of production (Place et al., 2006a).

However, the concentration of the residences around valley bottoms, where these activities of claiming swamps and forest for agriculture is running, is just exposed them to a large number of vectors and high level of malaria transmission (Ndenga et al., 2006). Accordingly, closer proximity to valley bottoms has been associated with increased malaria risk as these habitats enhanced *Anopheles* breeding sites and their microclimates being more suitable for prolong adult vector survival and parasite development; hence a 500-m threshold for these relationships has been demonstrated (Minakawa et al., 2004; Zhou et al., 2004; Ernst et al., 2009). Although these results indicated that malaria risk may cluster near specific land covers and topography, still some efforts are needed to identify such high risk areas, as even in these apparent hotspots, malaria-free households has been observed suggesting that environmental, socio-demographic, biological, and behavioral variables are important (Ernst et al., 2009).

The current malaria interventions (mainly ITNs) in the highlands have greatly reduced malaria prevalence in 2000s onwards (Stern et al., 2011; Chaves et al., 2012). Transmission has been interrupted in some areas (Zhou et al., 2011), which might lead to decrease in immunity of the residences (Githeko et al., 2012). With the reported environmental changes that alter malaria transmission, peoples in the highlands would be at high risk never like before if the interventions is not sustainable. Such present factors have already contributed to this scenario include reduced efficacy of ITNs, insecticide resistance and lack of proper use of ITNs (Protopopoff et al., 2008; Atieli et al., 2011; Zhou et al., 2011).

## Authors' contributions

Yousif E. Himeidan identified the idea, framed, drafted, and wrote up the manuscript, Eliningaya J. Kweka wrote up and reviewed the content. All authors read and approved the final version.

### Conflict of interest statement

The authors declare that the research was conducted in the absence of any commercial or financial relationships that could be construed as a potential conflict of interest.
